# Genome-wide identification, classification, and expression analysis of the JmjC domain-containing histone demethylase gene family in birch

**DOI:** 10.1186/s12864-021-08063-6

**Published:** 2021-10-28

**Authors:** Bowei Chen, Shahid Ali, Xu Zhang, Yonglan Zhang, Min Wang, Qingzhu Zhang, Linan Xie

**Affiliations:** 1 Key Laboratory of Saline-alkali Vegetation Ecology Restoration, Ministry of Education, College of Life Science, Northeast Forestry University, Harbin, 150040 China; 2 College of Life Sciences, Northeast Forestry University, Harbin, 150040 China; 3 State Key Laboratory of Tree Genetics and Breeding, Northeast Forestry University, Harbin, 150040 China

**Keywords:** Histone demethylation, JmjC domains, *BpJMJ* genes, Birch, Low-temperature stress, Seed germination, “Intracellular membrane”

## Abstract

**Background:**

Histone methylation occurs primarily on lysine residues and requires a set of enzymes capable of reading, writing, and erasing to control its establishment and deletion, which is essential for maintaining chromatin structure and gene expression. Histone methylation and demethylation are contributed to plant growth and development, and are involved in adapting to environmental stresses. The JmjC domain-containing proteins are extensively studied for their function in histone lysine demethylation in plants, and play a critical role in sustaining histone methylation homeostasis.

**Results:**

In this study, a total of 21 JmjC domain-containing histone demethylase proteins (JHDMs) in birch were identified and classified into five subfamilies based on structural characteristics and phylogenetic relationships among Arabidopsis, rice, maize, and birch. Although the *BpJMJ* genes displayed significant schematic variation, their distribution on the chromosomes is relatively uniform. Additionally, the *BpJMJ* genes in birch have never experienced a tandem-duplication event proved by WGD analysis and were remaining underwent purifying selection (Ka/Ks < < 1). A typical JmjC domain was found in all *BpJMJ* genes, some of which have other essential domains for their functions. In the promoter regions of *BpJMJ* genes, cis-acting elements associated with hormone and abiotic stress responses were overrepresented. Under abiotic stresses, the transcriptome profile reveals two contrasting expression patterns within 21 *BpJMJ* genes. Furthermore, it was established that most *BpJMJ* genes had higher expression in young tissues under normal conditions, with *BpJMJ06/16* having the highest expression in germinating seeds and participating in the regulation of *BpGA3ox1/2* gene expression. Eventually, *BpJMJ* genes were found to directly interact with genes involved in the “intracellular membrane” in respond to cold stress.

**Conclusions:**

The present study will provide a foundation for future experiments on histone demethylases in birch and a theoretical basis for epigenetic research on growth and development in response to abiotic stresses.

**Supplementary Information:**

The online version contains supplementary material available at 10.1186/s12864-021-08063-6.

## Background

In eukaryotes, the histone octamer is composed of two copies of H2A, H2B, H3, and H4 histones wrapped in 146-bp double-stranded DNA [1–4]. In *Arabidopsis thaliana*, numerous studies identified post-translational modifications (PTMs) of histones on peptides residue tails such as methylation, acetylation, phosphorylation, ubiquitination, and sumoylation [5, 6]. Different modification occurs at various histones of nucleosomes. For example, the lysine is methylated, acetylated, and ubiquitinated; arginine is methylated; and phosphorylation of threonine and serine. Histone methylation has powerful impacts on gene transcription and chromatin structure and could be pass on to the next generation through mitosis and meiosis [7].

Histone methylation is a reversible dynamic regulation process that involves methylation and demethylation. Histone methylation takes place primarily at the N-terminal arginine (R) and lysine (K) sites of histones H3 and H4, namely K4, K9, K27, and K36 for H3 and K20 for H4 [8]. S-adenosylmethionine was used as a methyl donor to target the N-terminal lysine (K) and arginine (R) of histone H3 and H4 through histone methyltransferases (HMTs) [9]. The protein family of SET or PRMT domains, which are primarily responsible for the methylation of histone lysine and arginine residues, has received considerable attention so far in plant histone methyltransferases studies [10]. Lysine residues can be monomethylated (Kme1), dimethylated (Kme2), or trimethylated (Kme3), while arginine can only be monomethylated (Rme1), symmetric dimethylated (Rme2s), or asymmetrically dimethylated (Rme2a) [11]. The methylation of histones has different impacts on gene expression; for example, H3K9 (H3K9me2/3) and H3K27 (H3K27me3) methylation are associated with transcriptional inhibition, while H3K4 (H3K4me2/3) and H3K36 (H3K36me3) methylation are associated with transcriptional activation [12]. These modifications serve as a kind of “histone code” for chromatin functions and transcriptional activities [13, 14]. As a result, histone alteration homeostasis is essential for plants’ normal growth and development [15].

Histone demethylation is primarily catalyzed by histone demethylase 1 (LSD1) and the protein containing the JmjC domain through amine oxidation and hydroxylation [16, 17]. Furthermore, the cofactors required by these two classes of histone demethylases show the different catalytic mechanisms: KDM1/LSD1 is a flavin adenine dinucleotide (FAD)-a dependent enzyme that can only remove single/double lysine residue methylation. However, JmjC domain-containing proteins that use Fe (II) and -ketoglutarate (KG) as cofactors will catalyze the removal of mono/di/tri-lysine residue methylation [18].

Proteins containing JmjC domain can be divided into 8 categories according to sequence similarity and catalytic specificity, namely KDM5/JARID1 group, KDM4/JHDM3 group, KDM3/JHDM2 group, JMJD6 group, JmjC domain-only group, KDM6/JMJD3 group, KDM2/JHDM1 group and PHF group [19]. However, only the first five subfamilies were found in the plant so far. Different JHDM subfamilies can catalyze the demethylation of different histones. For example, KDM5/JARID1 group proteins have been found to catalyze the demethylation of H3K4me1/2/3 [20–24], KDM4/JHDM3 family proteins are shown to demethylate histone H3K9me2/3 actively, and H3K36me2/3 [25–27], KDM3A/JHDM2 can demethylate H3K9me2 and H3K9me1 but not H3K9me3 [28], JMJD6 group can demethylate H3R2me2 and H4R3me2 [29], and JmjC domain-only subfamily can remove the methylation of H3K27me3 [30].

The *JHDM* gene family in different species shows various tissue-specific expression patterns. For example, in cotton, the *JHDM* gene family is mainly expressed in 20 and 30 days post-anthes (DPA) fibers [31]. In maize, the expression level in roots, stems, and leaves are relatively low, and also different genes exhibit various expressions in different tissues. For example, *ZmJMJ7* and *ZmJMJ17* are lower in silks and seedings, while *ZmJMJ16* is higher in shoot tips [32]; In soybeans, the *JHDM* genes show a relatively low expression level in four tissues (pods, pod seeds, roots, and seeds) but a higher expression in the flower, leaf, and shoot meristem [33]. Similarly, in *Rosa chinensis,* it was worth noting that most of the *JHDM* genes were higher expressed in reproductive tissues, especially in floral meristem and closed flowers, than vegetative tissues [34].

In *Arabidopsis thaliana*, the *AtJMJ14*, *AtJMJ15*, and *AtJMJ18* can demethylate H3K4 and participate in the regulation of flowering time and female gametophyte development [35–37]. *AtJMJ11/ELF6* (EARLY FLOWERING 6) and *AtJMJ12/REF6* (RELATIVE OF EARLY FLOWERING 6) play an opposite role in the flowering inhibition pathway, respectively [38, 39]. Additionally, *AtJMJ30* and *AtJMJ32* can regulate histone demethylation at FLC sites to prevent premature flowering under high-temperature treatment [40]. *AtJMJ20* and *AtJMJ22* can promote the expression of *GA3ox1/2* by changing the histone methylation status of H3K4 and H3R4, thereby affecting the germination of seeds [41]. In response to stress, *AtJMJ17* can directly bind to *OST1* (OPEN STOMATA 1) and adjust the expression level of *OST1* by removing the methylation of H3K4me3 in response to drought [42]. Overexpression of *AtJMJ15* in Arabidopsis directly results in a significant increase in salt tolerance [43]. It was also found in soybeans that salt treatment would increase the H3K4 trimethylation level and decrease the H3K9 methylation level of some genes [44]. In *Medicago truncatula*, *MtJMJ05* can produce alternative splicing in response to cold stress [45]. Above all, different abiotic stresses can modulate *JHDM* gene expression to control the growth and development of the plant under unfavorable conditions.

The present study is focused on a perennial woody tree (birch), which is mainly distributed in high latitudes and has important ecological and economic value. Previous studies on the *JHDM* gene family mainly focused on annual herbaceous plants, but little is known in perennial woody plants. Here, we performed a comprehensive analysis of the *JHDM* family genes in birch, including classification and architecture, chromosomal location, duplication events, Ka/Ks analysis and a functional analysis of cis-acting elements, tissue-specific expression patterns, and transcriptome profiling under different abiotic stress conditions.

## Results

### Identification and classification of the $JHDM$ gene family in brich

In order to identify all possible homologs of the *JHDM* gene family in birch, the full-length amino acid sequences of the JHDM proteins determined in Arabidopsis, rice, and maize were performed to query the protein sequence database of the *Betula pendula* genome with blastp program. After de-forgery and de-redundancy, 21 *JHDM* genes were identified and named *BpJMJ01* to *BpJMJ21* (Table S1).

**Fig. 1:**
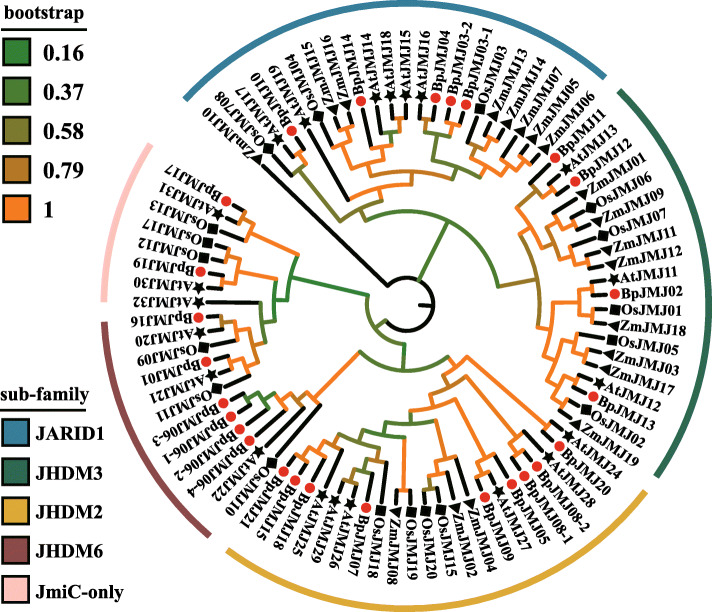
Phylogenetic analysis of JHDM proteins in maize, rice, Arabidopsis, and birch. The rooted neighbor-joining (NJ) phylogenetic tree of JHDM family was clustered with bootstrap values shown for each clade in a different color. The BpJMJ family have been highlighted for each group with a red $\mathrm{circle}$. Five clades are marked as JARID1, JHDM3, JHDM2, JHDM6, and JmjC-only, respectively

To examine the evolutionary history of these protein families in these four species and to establish the phylogenetic relationship among *JHDM* family genes, the phylogenetic tree was constructed with MEGA6.0 based on the neighbor-joining algorithm with full-length JHDM protein sequences of birch (21), Arabidopsis (21), rice (20), and maize (21) (Fig. 1). Based on the branching characteristics and bootstrap values in the phylogenetic tree, a total of 21 *BpJMJ* genes are classified into five major subfamilies: *JARID1/KDM5* (4 genes), *JHDM3/KDM4* (4 genes), *JHDM2/KDM3* (8 genes), *JHDM6* (3 genes) and *JmjC-domain only* subfamily (2 genes), respectively (Table S1). Interestingly, more than 70% of BpJMJ proteins share the highest protein sequence similarity with Arabidopsis homolog (Blastp *P*-value = 0) (Table S2), showed that the birch is a dicotyledonous tree species with 5 JHDM superfamilies’ simultaneously and has a closer genetic relationship with Arabidopsis, and away from rice and maize as monocotyledonous species.

Furthermore, the 21 filtered JHDM amino acid sequences were analyzed in the Expasy website, including isoelectric point (PI) and molecular mass (kDa). The results show that the amino acid length of BpJMJ proteins ranges from 404 amino acids of BpJMJ19 to 2205 amino acids of BpJMJ13. In addition, the molecular mass ranges from 45,702.96 to 246,534.29 Da, indicating that the protein properties of JHDMs in birch have a wide change (Table S1).

### The structures of identified *JHDM* genes and protein in birch

**Fig. 2:**
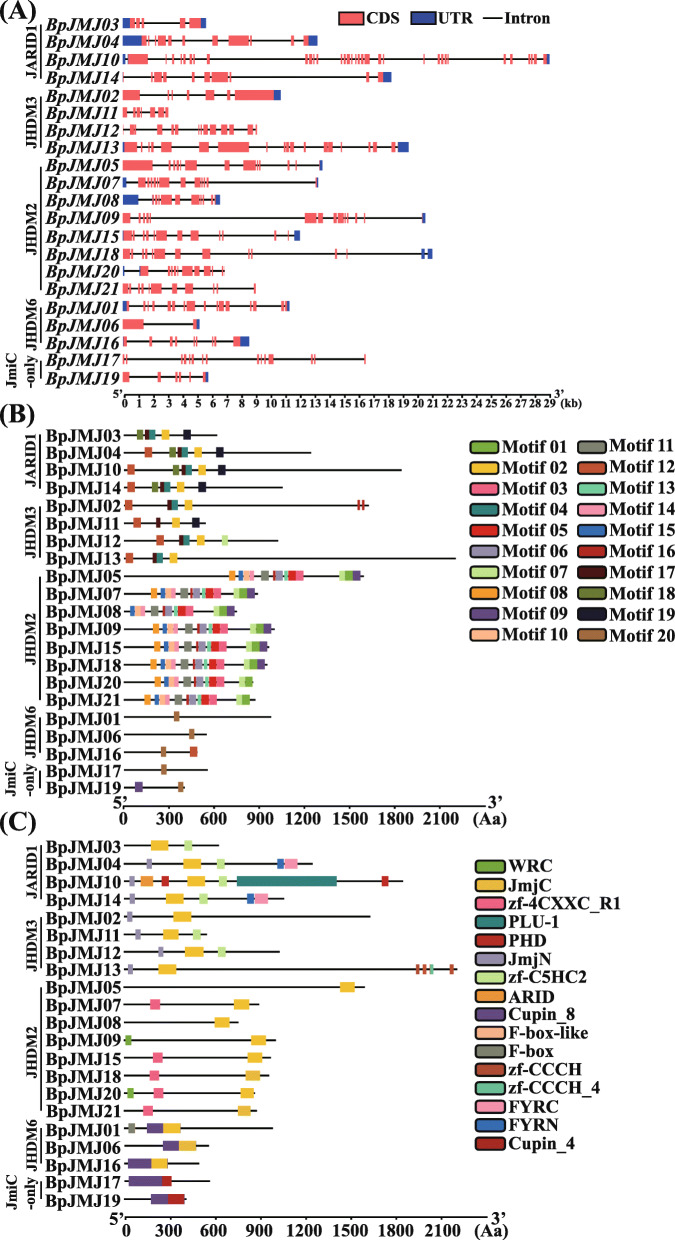
Schematic structure of *JHDM* genes in birch. The $\mathrm{nucleic}$ or protein sequence is represented as a black line; the N terminus is left for each. The length of each *JHDM* gene can be estimated using the scale at the bottom. **A** Exons and introns are indicated by red boxes and single lines, respectively. The boxes in the dark blue represent the untranslated regions (UTRs). **B** Different classes of motifs are shown with boxes in different colors. Only the top 20 most frequently occurring motifs are displayed. **C** Conserved domains are identified in each *BpJMJ* genes subfamily. The location and size of domains are shown by different color rectangles annotated in the legend

The structural heterogeneity of individual *BpJMJ* genes by exon-intron and corresponding domain architecture was investigated, and the variation among them is unprecedented. The average exon count for *BpJMJ* family is 13.52 per gene; the exon count varies considerably, ranging from 3 exon of *BpJMJ06* to 36 exon of *BpJMJ10* (Fig. 2A). The *BpJMJ10* gene has the highest exon (36) and the most extended nucleic acid sequence in the *JARID1/KDM5* subfamily (29,078 bp). The *BpJMJ07*, *BpJMJ08*, and *BpJMJ17*, *BpJMJ18* share a similar exon-intron structure in *JHDM2/KDM3*, except for *BpJMJ05*, *BpJMJ09*, and *BpJMJ20*. Furthermore, transposon insertion is likely to cause *BpJMJ* genes with very long introns, such as *BpJMJ08, BpJMJ09, BpJMJ10, BpJMJ14*, and *BpJMJ17*, which may influence the expression of the corresponding gene and cause variable splicing.

Further, the JHDM protein sequences were submitted to the MEME website with the default parameters, and up to 20 motifs (motif01-motif20) with a width between 6 and 50 were analyzed. The width, sites, and E-value of predicted conserved motifs for each BpJMJ protein are provided in Fig. S1, and Table S3. According to the sequence motif similarity, BpJMJ family could be classified into three major groups. The first group consists of the JARID1 and part JHDM3 families with 4.75 motifs on average, and the second group consists of a single family of JHDM2 proteins with 13 motifs on average. The third group, including JHDM6 and JmjC-domain family, shares an average of 1.4 motifs (Fig. 2B). The result is similar to the classification of JHDM families in the phylogenetic tree.

To know more about the BpJMJ protein domain architecture diversity. All BpJMJ protein sequences were submitted in the PFAM and SMART websites with default parameters. Based on the evolutionary relationship of JHDM family in the previous phylogenetic tree, the BpJMJ family can be divided into five subfamilies, including (a) JARID1/KDM5 subfamily (4), (b) JHDM3/KDM4 subfamily (4), (c) JHDM2/KDM3 subfamily (8), (d) JHDM6 subfamily (3) and (e) JmjC-only domain subfamily (2) (Fig. 1). In the (a) JARID1/KDM5 subfamily and (b) JHDM3/KDM4 subfamily, except that BpJMJ03 lacks a JmjN domain and BpJMJ13 lack a zf-C5H2, all other protein sequences share similar domain architecture, mainly including JmjN, JmjC, and zf-C5H2. In addition, for the JARD1 subfamily, BpJMJ04 and BpJMJ14 also have additional FYRN and FYRC domains, which may have the ability to bind chromatin in the trithorax/ALL1 protein family [19]. BpJMJ10 has ARID, PHD, and PLU domain which shows high similarity with ZmJMJ10 in maize (Fig. 2C, and Table S4). And also, the BpJMJ13 has four zf-CCCH domains at the N-terminal as well, which is a short zinc-binding domain that has the pattern of three cysteines and one histidine to coordinate the zinc ion. In the (c) JHDM2/KDM3 subfamily, a total of 8 proteins mainly share only the JmjC domain and zf-4CXXC_R1 domain, but not the JmjN domain. All members, in both the (d) JHDM6 subfamily and (e) JmjC-domain only subfamily, share the same Cupin-8 domains, but there will be another JmjC domain in (d) JHDM6 subfamily and Cupin-4 in (e) JmjC-domain subfamily only (Fig. 2C). In summary, the JmjC domain is the most widespread architecture of the protein domain, which belongs to the Cupin superfamily and maybe a protein hydroxylase that catalyzes histone lysine demethylation [46].

In addition, two cofactors, Fe (II) iron and alpha-KG, mainly interacting with five key amino acid residues, are essential sites for the process of histone demethylation. The first group, including three amino acid residues (His188, Glu190, and His276), is indispensable for Fe (II) iron-binding, and the second group including two amino acid residues (Thr185 and Lys206) required for αKG binding.

To further confirm whether these conserved residues bound by cofactors had diverged among the BpJMJ proteins, all JHDM proteins from Arabidopsis, rice, and birch were performed with homologous alignment. The JHDM proteins can be divided into 2 categories depending on the retained amino acid residues of the alignment results: the first category includes the JARID1/KDM5 and the JHDM3/KDM4 subfamily, primarily His (H), Glu (E), and His (H) for Fe (II) binding, and the JHDM2/KDM3, JHDM6 and JmjC-only domain subfamily, mainly Phe (F) and Lys (K) for alpha-KG binding (Fig. S2); the second category includes JARID1/KDM5 subfamily and JHDM3/KDM4 subfamily, mainly His (H), Asp (D) and His (H) for Fe (II) binding, and in JHDM2/KDM3, JHDM6 and JmjC-only domain subfamily, mainly Thr (T) and Lys (K) for alpha-KG binding (Fig. S3).

These two categories of conserved amino acid residues for Fe (II) and α-KG binding can both exert histone demethylation function [47, 48]. The alignment results show that the most conserved amino acid residues for Fe (II) and α-KG binding sites among JHDM proteins in each category are same. However, there are still some JHDM protein whose amino acid residues are variable. For example, in the protein sequence of BpJMJ03, the first and third amino residues of the Fe (II) binding site are changed from His (H) to K (Lys) and Y (Tyr) separately, in BpJMJ01 and BpJMJ06, the first amino acid residue of the binding site of alpha-KG is changed from T (Thr) to S (Ser) and A (Ala) (Fig. S2, and Fig. S3). Taken together, the cofactors binding sites (Fe (II) and α-KG) composed of the five amino acid residues are quite important for the function of histone lysine demethylation in birch.

### Chromosomal location and gene duplication of *JHDM* gene family in birch

**Fig. 3:**
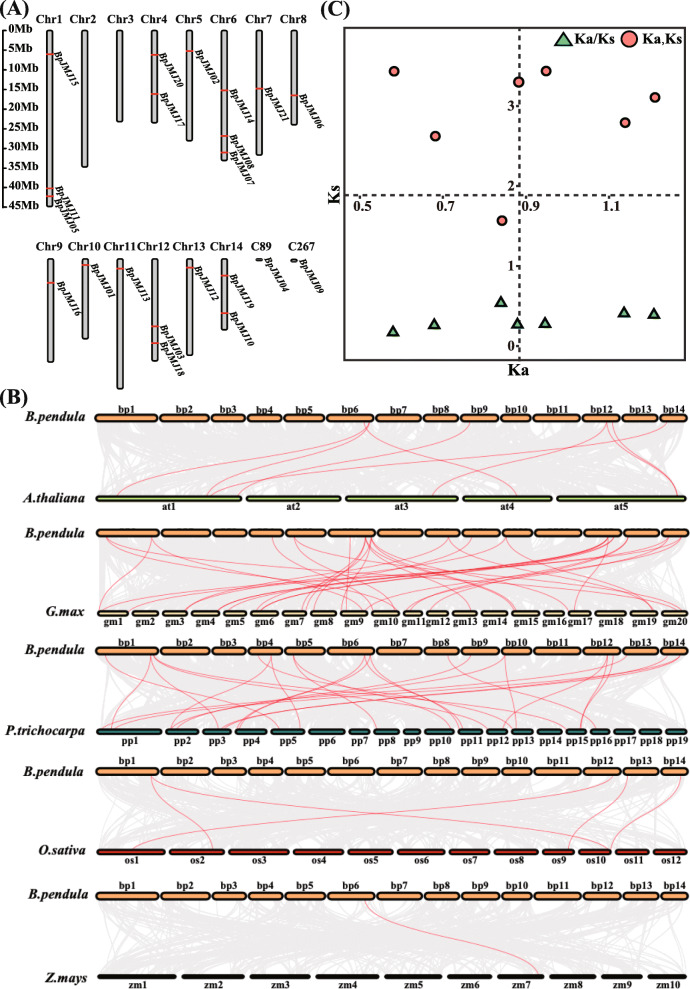
Chromosomal localizations, gene duplication events, and Ka/Ks analysis of *BpJMJ* genes. **A**. The distribution of the 21 *BpJMJ* genes was mapped on the 14 chromosomes and 2 contigs. The chromosome numbers are indicated at the top of each vertical gray bar. The gene names on each chromosome’s right side correspond to the approximate locations of each *BpJMJ* gene. The scale on the left is in megabases. **B** The synteny analysis of the *BpJMJ* genes between birch and five representative plant species. Grey lines indicate the collinear blocks between birch and other plant genomes, while the red lines indicate the syntenic *BpJMJ* gene pairs. The plant named different prefixes ‘*B. pendula*’, ‘*A. thaliana*’, ‘*G. max*’, ‘*P. trichocarpa*’, ‘*O. sativa*’ and ‘*Z. mays*’ represent *Betula. pendula*, *Arabidopsis thaliana*, *Glycine max*, *Populus trichocarpa*, *Oryza sativa,* and *Zea mays*. **C** The ratio between Ks and Ka for paralogous *BpJMJ* gene pairs in birch

The physical position of 21 *BpJMJ* genes, 19 of which are more evenly spread over the whole chromosome, was explored to elucidate the spatial distribution of the *BpJMJ* genes family in each chromosome. Unexpectedly, the other two genes (*BpJMJ04* and *BpJMJ09*) were annotated on the contigs, which are not assembled into the genomic chromosomes (Fig. 3A).

Moreover, gene duplication events are frequently associated with the plant evolution process and contribute to the gene family’s expansion [49, 50]. Here, the evolutionary patterns of the *BpJMJ* genes were surveyed in MCScanX software, and amazingly we found that there are no tandem duplications for *BpJMJ* genes (Table S5), speculating that the *BpJMJ* genes family in the long evolution of brich has never experienced a tandem-duplication event.

To explore the potential evolution relationship and further compare the *JHDM* gene family collinearity among different species, a comparative analysis between the BpJMJ protein and homologues from the other five representative plants, including *Arabidopsis thaliana*, *Glycine max*, *Populus trichocarpa, Oryza sativa*, and *Zea mays*, was performed. The results show that there are 6(28.5%), 15(71.4%), 16(76.2%), 4(19.0%), and 1(4.8%) *BpJMJ* proteins show high homology to the members from the other five species, respectively (Fig. 3B). Conclusively, the *JHDM* gene family in birch has more collinearity with dicotyledonous plants (*Arabidopsis thaliana, Glycine max, and Populus trichocarpa*), but shows a little intersection with monocotyledonous plants (*Oryza sativa* and *Zea mays*), indicating that the *JHDM* genes family has undergone evolutionary divergence in monocotyledonous and dicotyledonous plants.

The Ka/Ks ratios of the paralogous genes measured in the *JHDM* gene subfamily are all less than 1 (Fig. 3C), indicating that the *JHDM* gene family undergoes purifying selection during evolution, which helps to understand the evolution of the birch *JHDM* gene family in dicotyledonous plants. Furthermore, the divergent period of the *JHDM* gene family in birch (woody plant) has been traced back to 52.19 MYA (Table S6), even earlier than in Arabidopsis (herb) (9.6–16.1 MYA) [51].

### The cis-acting regulatory elements in the promoter of *JHDM* genes in birch

**Fig. 4:**
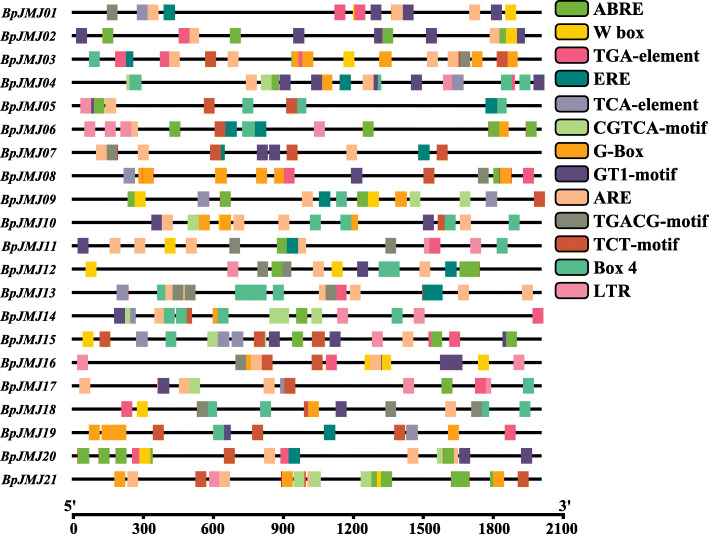
Cis-elements in the promoter sequences of the 21 *BpJMJ* genes. Distribution of cis-acting elements related to hormone, light, and stress in the promoter sequences of the 21 *BpJMJ* genes. Putative ABRE, W-box, TGA-element, ERE, TCA-element, CGTCA-motif, G-box, GT1-motif, ARE, TGACG-motif, TCT-motif, Box 4, and LTR core sequences are represented by different symbols as indicated in legend at the right. The 2 kb sequences upstream of the initiation codon (ATG) of the *JHDM* genes can be estimated using the scale per 300 bp above

To elucidate the *JHDM* gene family regulatory mechanism about abiotic or biotic stress in birch, the genomic sequence of 2 kb upstream promoters of birch *JHDM* family genes was used to query the Plant Care database to search for cis-regulatory elements (CREs). The 13 classes of CREs related to hormone (ABRE, CGTCA-motif, TGACG-motif, ERE, and TCA-element), light reaction (G-box, GT1-motif, TCT-motif, and Box-4), and stress (ARE, W-box, and LTR) were detected (Fig. 4, and Table S7). Each predicted CRE exists in at least 9 *JHDM* genes, and each gene has at least 6 CREs distribution (Fig. S4). In detail, hormone-related CREs, including ABRE (abscisic acid-responsive), CGTCA-motif (MeJA-responsive), TGACG-motif (MeJA-responsive), TGA (salicylic acid-responsive), ERE (ethylene-responsive) and TCA-motif (salicylic acid-responsive), has largest proportion and distributed in 20, 17, 14, 12 and 9 *BpJMJ* genes, respectively. Further, followed by CREs related to light response, including G-box, GT1-motif, TCT-motif, and Box-4, are distributed in 18, 17, 16, and 14 *JHDM* genes, respectively. The last is stress-related CREs, including ARE (anaerobic induction), W_box (wounding and pathogen responsive), and LTR (low-temperature responsive) are distributed among 19, 13, and 10 *JHDM* genes, respectively (Fig. S5). Various environmental variables, such as hormones, light, and low-temperature stress, disrupt the transcriptional control of *BpJMJ* genes.

### Transcriptome analysis and role of *JHDM* genes in seed germination of birch

**Fig. 5:**
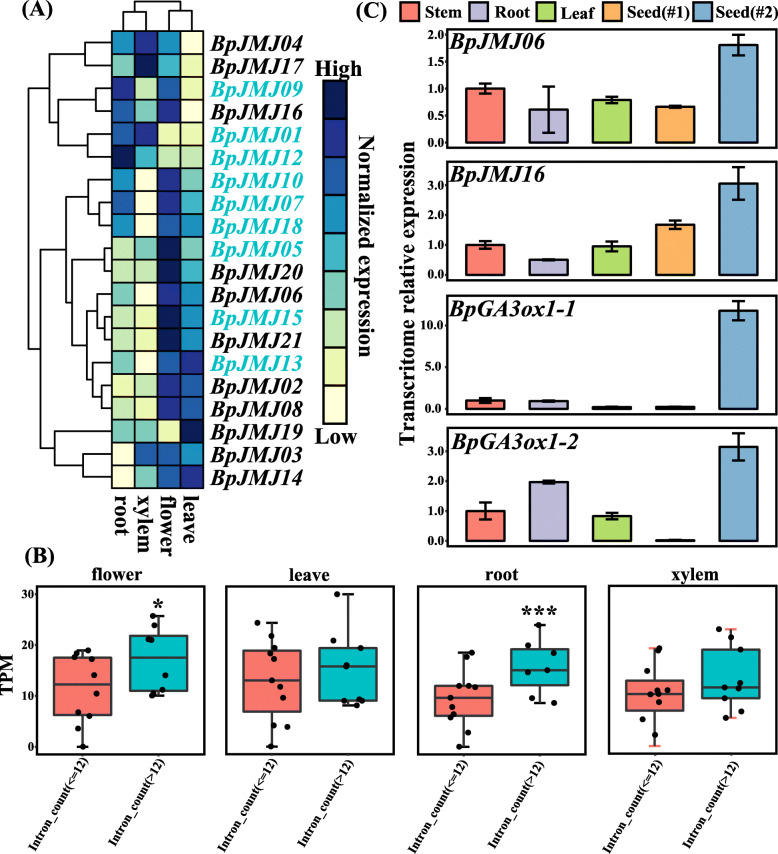
Tissue-specific expression profiles of *BpJMJ* genes in birch. **A**. The hierarchical clustering of expression profiles of 21 *BpJMJ* genes in four tissues. The color scale representing gene TPM (Transcripts Per Million) is shown on the right. The cell in yellow represents low gene expression, and the blue one indicates high gene expression. The different tissues are noted on the bottom of each line. Cluster dendrograms are shown on the left and above, respectively. **B**. The tissue-specific expression pattern of *BpJMJ* genes and their positive correlation with intron number. The significance of the difference was determined using the *t.test*. **C**. Tissue-specific relative expression of *BpJMJ06/16* and *GA3ox1/2* examined by qRT-PCR. The qRT-PCR data was normalized using the birch *actin* gene and are shown relative to the stem. The X-axes show different tissues (stem, root, leaf, ungerminated seeds (Seed#1), and germinating seeds (Seed#2)), and y-axes are scales of relative expression level. Bars represent standard deviations (SD) of three technical replicates

To better understand the potential roles of the *BpJMJ* genes family in plant growth and development, a comprehensive expression analysis was accomplished based on tissue-specific transcriptome data of two-year-old birch released by Chen et al. 2019 [52]. It can be seen from the heatmap that, except for *BpJMJ11*, the expression of the other 20 *BpJMJ* genes in various tissues is quite different. The majority of *BpJMJ* genes are heavily expressed in young tissues, such as birch roots and flowers (Fig. 5A, and Table S8). All members of the *JHDM2* subfamily, as well as the vast majority of the *JHDM3* subfamily (*BpJMJ02, BpJMJ1*3, and *BpJMJ15*)*,* the *JARID1* subfamily (*BpJMJ03* and *BpJMJ14*), and the *JHDM6* subfamily (*BpJMJ06* and *BpJMJ16*) are highly expressed in floral tissues and partially in leaf tissues. Interestingly, the JmjC-only subfamily has a high level of expression in mature tissues. For instance, *BpJMJ17* is highly expressed in the xylem, while *BpJMJ19* is highly expressed in the leaves.

Furthermore, the *JHDM* genes have a huge diversity in their gene structures within the same subfamily, which is often displayed in the number of introns (Fig. 2A). Previous research has shown that increasing the number of introns will improve mRNA stability and promote gene expression [53]. As a result of evaluating the relationship between the number of introns in each *JHDM* family gene and their expression levels in various tissues, the number of introns in the *JHDM* gene was strongly positively correlated with gene expression in the roots and flowers (Table S9). The importance of variations in the expression of *JHDM* genes with different numbers of introns was investigated, and it was revealed that the *JHDM* gene with more introns expressed higher in the young tissues (Fig. 5B). For example, the *BpJMJ07*, *BpJMJ09*, *BpJMJ10*, and *BpJMJ18,* with 14, 13, 15, and 15 introns respectively, are relatively highly expressed in roots and flowers.

On the other hand, previous studies have shown that histone modifications can regulate plant growth and development in response to changes in the external environment. Cho et al. (2007) [41] have reported that *JMJ20/22* of Arabidopsis could regulate the expression of *GA3ox1/2* in response to light, thereby affecting seed germination. The *BpJMJ06/16* in birch are the exact homologous genes of *AtJMJ20/22*. The expression of *BpJMJ06/16* was quantified using qRT-PCR in various tissues, including stem, root, leaves, ungerminated seeds, and germinating seeds. The results show that the *BpJMJ06/16* were highly expressed in germinating seeds and maintained a relatively low expression in other mature tissues (Fig. 5C). Additionally, the *BpGA3ox1/2* shows a similar tissue-specific expression pattern with *BpJMJ06/16*, suggesting that *BpJMJ06/16* could promote the germination of birch seeds which may affect the expression of *BpGA3ox1/2* by regulating histone methylation modification.

### Transcriptome analysis of birch *JHDM* gene family in response to abiotic stress

**Fig. 6:**
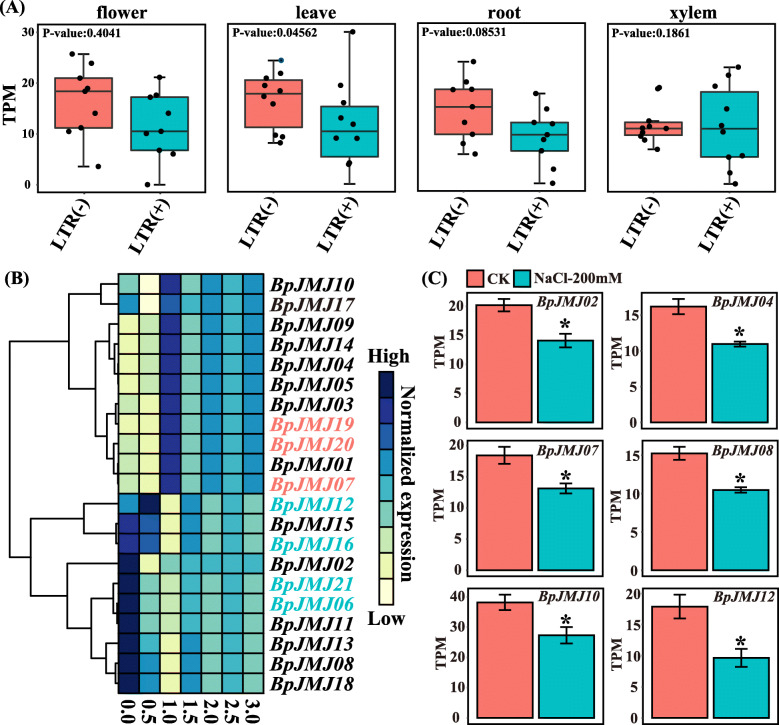
The expression profiles of *BpJMJ* genes response to abiotic stress. **A**. The tissue-specific expression pattern of *BpJMJ* genes with/without LTR elements. The *t. test* was used to determine the significance of the difference, and the dashed box indicates the two groups of genes with larger differences for subsequent verification. **B**. The *BpJMJ* gene expression with cold stress. The color scale representing gene TPM (Transcripts Per Million) is shown on the right. The cell in yellow represents low gene expression, and the blue one indicates high gene expression. The horizontal axis represents the cold treatment time of birch, including 0.5 h, 1.0 h, 1.5 h, 2.0 h, 2.5 h, and 3.0 h, and the vertical axis represents 21 *BpJMJ* genes clustered by their TPM. **C**. The *BpJMJ* gene expression **with salt stress.** The *JHDM* genes with significant differences are shown above. The red bar represents control, the green bar represents 200 mM NaCl salt treatment, and the ordinate represents TPM. “*” means that the *P*-value calculated by the t-test is less than 0.05

To investigate the *JHDM* gene family’s expression in response to abiotic stress. The Spearman-correlation analysis between the number of 13 cis-acting elements in the *BpJMJ* gene promoter regions and their expression in different tissues revealed that LTR exhibits a significant correlation among the flowers, leaves, and roots (Table S10). Furthermore, *BpJMJ* genes with LTR elements have significantly lower expression in birch leaves than non-LTR *BpJMJ* genes (Fig. 6A), implying that the LTR factor will react to the external low temperature by down-regulating gene expression.

To further validate this hypothesis, the cold-treated (6 °C) transcriptome of the two-month-old birch from the State Key Laboratory of Northeast Forestry University was used for analyzing the gene expression pattern. The findings revealed that low-temperature stress-induced all *JHDM* genes in birch with no quantitative preference, but there were two opposing expression patterns: (I) *JHDM* gene up-regulation (11 genes) and (II) *JHDM* gene down-regulation (10 genes) in 1 h (Fig. 6B, and Table S11). Surprisingly, genes containing LTR elements, such as *BpJMJ12, BpJMJ16, BpJMJ21,* and *BpJMJ06*, were down-regulated, while genes without LTR elements, *BpJMJ07*, *BpJMJ19,* and *BpJMJ20*, were high expression under cold stress conditions (Fig. 6B). Taken together, the *BpJMJ* gene containing LTR elements could be down-regulated in response to low temperature.

On the other hand, in the *JARID1/KDM5* subfamily and *JmjC-domain only* subfamily, the genes included *BpJMJ03, BpJMJ04, BpJMJ10, BpJMJ14, BpJMJ17*, and *BpJMJ19* are belong to the up-regulated genes, the *JARID1/KDM5* subfamily genes are involved in the H3K4me1/2/3 demethylase. In the *JHDM3/KDM4* subfamily, all genes (*BpJMJ02, BpJMJ11, BpJMJ12,* and *BpJMJ13*) are inhibited by cold stress. The above-mentioned two *JHDM* gene expression patterns may eventually result in the down-regulation of the whole genome H3K4me1/2/3 level and the up-regulation of the H3K9me2/3 level. There are differences in expression patterns with low-temperature stress in the *JHDM2/KDM3* subfamily and the *JHDM6* subfamily. Four genes (*BpJMJ05, BpJMJ07, BpJMJ09, BpJMJ20*) and one gene (*BpJMJ01*) are up-regulated, and the other four genes (*BpJMJ08, BpJMJ15, BpJMJ18, BpJMJ21*) and 2 genes (*BpJMJ16*, *BpJMJ06*) expressions are inhibited by cold stress, which may lead to down-regulation and up-regulation of H3K9me1/2 and H3K27me2/3 in the whole genome of birch. Above all, the gene expression of the JHDM family is severely affected by low-temperature stress.

Moreover, the salt treatment can also affect the expression of the *JHDM* subfamily. By sequencing the transcriptome of the leaves after salt treatment, it was found that a total of 7 *BpJMJ* genes expression was inhibited by 200 mM salt treatment, which was distributed among *JARID1* (*BpJMJ04, BpJMJ10*), *JHDM3* (*BpJMJ02, BpJMJ12*), and *JHDM2* (*BpJMJ07, BpJMJ08*) subfamily (Fig. 6C, and Table S12). There were no significantly up-regulating for *BpJMJ* genes during salt treatment (Fig. S6). In summary, salt treatment could have inhibited the expression of some *BpJMJ* genes in leaves of birch.

### Co-expression of low-temperature stress genes in the $BpJMJ$ gene family

**Fig. 7:**
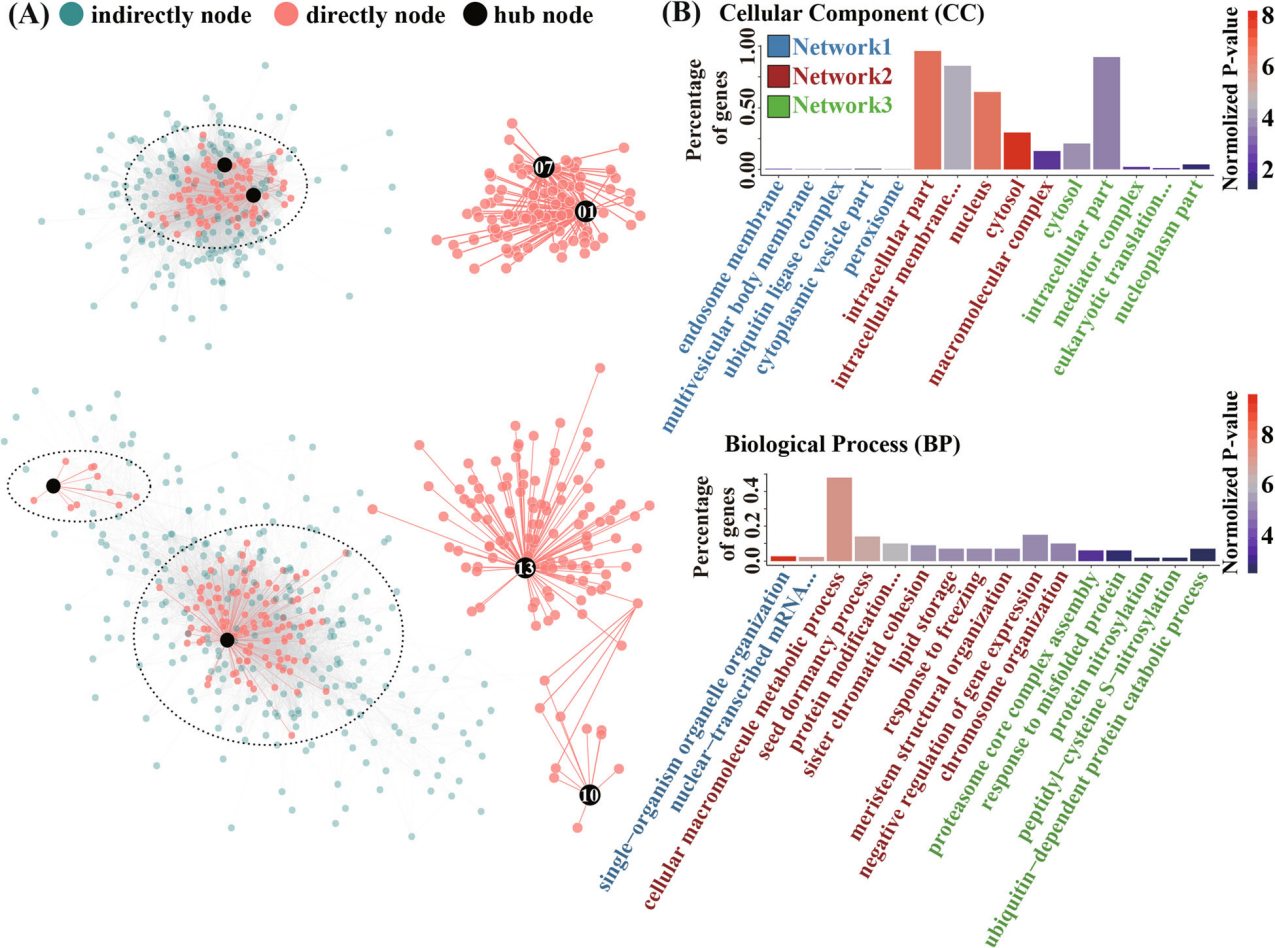
The co-expression network for the low-temperature response *BpJMJ* genes and GO enrichment analysis. **A**. Co-expression network. On the left is the complete gene expression network, and on the right is the network of genes directly related to *BpJMJ* genes. The green dots indicate genes that are not directly related to *BpJMJ* genes, and the red dots indicate genes directly related to *BpJMJ* genes. **B**. GO enrichment analysis. The ordinate represents the abundance of enrichment, the color of the histogram represents the -log10(*P*-value), the terms in blue, red and green color represent the GO enrichment result of *BpJMJ*-directly genes from network1, network2 and network3, respectively

To further study the mechanism of the birch *JHDM* gene family in response to low-temperature stress, the 16 transcriptome profiles under cold stress were used to construct a gene co-expression network by WGCNA. There were a total of 28 expression pattern modules identified (Fig. S7). A total of 6 *BpJMJ* genes equally participate in three co-expression network modules with a weight threshold of Pearson correlation coefficient (greater than 0.1). The largest co-expression network (network1) contains 2020 genes and 851,957 interactions, with 877 (43.42%) genes having close links to BpJMJ04 and BpJMJ20 (Fig. S8). The second interaction network (network2) comprises 377 genes and 7663 interactions, with 81 genes (21.49%) related to BpJMJ01 and BpJMJ07. Lastly, the smallest co-expression network (network3) consists of 255 genes and 9663 interactions, with 112 genes (43.92%) related to BpJMJ10 and BpJMJ13 (Fig. 7A).

Furthermore, gene ontology (GO) analysis was performed on the co-expression genes directly related to the *BpJMJ* genes in each of the three networks. By dissecting the co-expressed genes, 600 co-expression genes were found to show a significant relationship with GO term “intracellular part” (Fig. 7B), of which 156 genes were in the cellular components of network2 and network3, and 444 genes are involved in intracellular transport in network1. The trait of resistance to cold has been identified in particular concerning intracellular signal transduction, which can effectively improve the cold resistance of plants by promoting the stability of intracellular membranes [54]. In addition, the term “calcium ion channels” was found in go enrichment result of the network1, which has been extended to have a response to cold stress (Fig. S9). Similar to network3, some terms related to the proteasome and ubiquitin degradation were also detected (Fig. 7B). Lastly, some co-expression genes were shown to be related to seed dormancy, lipid storage, chromosomal reorganization, response to freezing, and inhibition of gene expression in network2.

## Discussion

Histone methylation is a post-translational modification of specific amino acids that regulates gene expression by modulating transcription factor accessibility to target genes. Histone methylation is linked to a variety of complex biological functions, including heterochromatin formation and transcriptional regulation. Histone methylation is implicated in the repression or activation of gene expression. The methylation and demethylation of histone modifications have been linked to chromatin state in plants, and this has been shown to influence plant growth and development. Histone demethylases with the JmjC domain, which is strongly conserved in plants, play an important role in preserving histone methylation homeostasis in vivo. In animals and plants, proteins with the JmjC domain belong to a large family of histone lysine demethylases that play an important role in histone modification, which is an important aspect of epigenetics. In this study, a comprehensive group of 21 *JHDM* genes was identified and characterized from *Betula pendula* genome, including phylogenetic tree, gene structure, domain and motif architecture, chromosome location, duplication events, Ka/Ks analysis, expression profiles and WGCNA analysis.

The phylogenetic study revealed that the *JHDM* gene family is highly conserved in both perennial woody plants and annual herbs, with 21 *BpJMJ* genes divided into five subfamilies: *JARID1*(4), *JHDM3*(4), *JHDM2*(8), *JHDM6*(3), and *JmiC-only* (2) in birch. Furthermore, since the *JHDM* gene family in birch has not undergone large-scale gene duplication events, its number of genes is quite similar to those of Arabidopsis (21) [55], rice (20) [56], and maize (19) [32], demonstrating the *JHDM* gene family’s significance in plant evolution. However, gene collinearity analysis proved that, compared with monocots, there are more homologous *JHDM*s among birch and dicots. This may cause by the loss of gene family, such as the absence of *JHDM6* and *JmiC-only* subfamily in maize [32], which gives rise to the evolutionary divergence of the *JHDM* gene family in monocots and dicots.

We also investigated the composition of birch JHDM proteins and discovered some preliminary structural characteristics. Except that BpJMJ03 lack of a JmjN domain and BpJMJ13 lack of a zf-C5H2, the JARID1/KDM5 and JHDM3/KDM4 subfamilies share identical domain architecture, with JmjN, JmjC, and ZF-C5H2 being the most common. BpJMJ04 and BpJMJ14 both have additional FYRN and FYRC domains for the JARD1 subfamily, which may have the capacity to bind chromatin in the trithorax/ALL1 protein family [19, 57]. Through the interaction of FYRC domains with transcription factors, *AtJMJ14* has been shown to play a role in Arabidopsis flowering time regulation [35, 58]. A total of 8 proteins in the JHDM2/KDM3 subfamily share only the JmjC domain but not the JmjN domain. All genes share Cupin-8 domain in the JHDM6 subfamily and JmjC-domain only subfamily, but there will be another JmjC domain in the JHDM6 subfamily and Cupin-4 in the JmjC-domain only subfamily. The JmjC domain, which belongs to the Cupin superfamily and maybe a protein hydroxylase that catalyzes histone lysine demethylation, is the most common protein domain architecture.

The divergence time for paralogous *BpJMJ* genes was furtherly calculated. The divergence time for paralogous gene pairs indicates that their divergence occurred much earlier than that of *A. thaliana* (9.6–16.1 MYA). Generally, if the value of Ka/Ks is < 1, it indicates gene pairs may have evolved from purifying selection (also called negative selection); Ka/Ks = 1 suggests neutral selection, while Ka/Ks > 1 means positive selection [51].

The *JHDM* gene family has been shown to have a variety of functions, including involvement in growth and development processes such as cell division and differentiation, such as flowering [59, 60], photoperiod [59], and hormone stress [61], as well as the ability to reverse methylation at multiple sites, including H3K4, H3K9, and H3K27. Recent studies have reported that several histone modifications, including H3K4me3, H3K9ac, H3K9me2, H3K23ac, H3K27ac, H3K27me3, and H4ac, along with DNA methylation response to abiotic stresses, such as drought stress, salt stress, and temperature fluctuations [62, 63]. Furthermore, similar research in maize has confirmed that most *ZmJMJ* gene expressions were promoted by heat stress [32]. To further investigate the response of the *JHDM* gene family to abiotic stress in birch, the transcriptome of low-temperature treatment was used to reveal the *BpJMJ* gene expression pattern. After being exposed to low-temperature stress for 1 h, the expression of 11 *BpJMJ* genes was significantly up-regulated, whereas the expression of the other ten genes was inhibited. These two expression categories have some links with the class of the *JHDM* gene subfamily. For example, the expression of all genes in the *JARID1/KDM5* subfamily is up-regulated by cold stress, while the expression of all genes in the *JHDM3/KDM4* subfamily is down-regulated under cold stress [64]. Similarly, the expression of the *JARID1* family can be up-regulated with high-temperature stress in maize, so it is speculated that temperature-induced stress can affect the expression of JARID1 subfamily genes among different species.

The Low temperature-responsive elements (LTRE), the basic sequence is ‘CCGAAA’, act as cis-element involved in low-temperature responsive. LTREs were also observed with very relative low frequencies in gene promoter sequences that are functionally involved in condition-specific heat stress and with hexamer (CCGAAA) signature [65]. However, so far, the research on the response mechanism of LTRE has mainly focused on heat shock proteins (HSPs). Previous studies have shown that the two elements, heat shock element (HSE) and LTR, have a synergistic effect in the response of HSPs in green macroalga to cold stress [66]. However, there are relatively few studies on LTRE in other genes. Previous studies on *JmjC* genes found that at least in maize, cotton and cabbage, its gene expression will be affected severely by low-temperature stress. Therefore, the correlation between the LTR element in the promoter region of the *BpJMJ* gene and its expression under low-temperature stress was performed to analysis, and found that the existence of LTREs may inhibit the expression of *BpJMJ* genes under low-temperature conditions, with a similar phenomenon in cotton [32].

Eventually, through sequence alignment and phylogenetic relationship analysis, *BpJMJ06/16* were found to be homologous genes to Arabidopsis *JMJ20/22*. It was documented that it could affect seed germination by modulating the dynamic balance of H3K9me3 and H3K4me3 in response to the external environment. Complete loss of PRC2 activity leads to derepression of seed maturation and dormancy genes at the seedling stage and subsequently compromises germination [67]. Here, in order to analyze whether *BpJMJ06/16* plays a role in the germination of birch seeds, the gene expression in different tissues was verified by qRT-PCR. It was found that the expression level in the mature tissues was relatively low, but it was highly expressed in germinating seeds, suggesting that in young tissues, it may be necessary to enhance the expression of genes that activate seed germination by increasing the modification of H3K4me3. The genes, *GA3ox1* and *GA3ox2*, have been expressed mainly in the embryonic axis and directly regulated by *JMJ20* and *JMJ22* in Arabidopsis [41]. Here, the expression of *GA3ox1/2* in birch was detected by qRT-PCR, which shows a similar tissue-specific expression pattern with *BpJMJ06/16*, so it is speculated that it may have the same regulatory mechanism as in Arabidopsis.

## Conclusion

In this study, we identified a total of 21 *JHDM* genes in birch, which were evenly distributed between 12 chromosomes and two contigs. Based on amino acid similarities, these *BpJMJ* genes were divided into five subfamilies. Despite the wide structural variation in exon/intron, functional domains and amino acid residues were more conserved among genes in the same subfamily. Phylogenetic clustering and collinearity analysis revealed that the *BpJMJ* genes have a closer relationship with dicots, and its divergence occurs even earlier than Arabidopsis and other cruciferous plants. The transcriptome profiles showed that the *BpJMJ* gene is mostly expressed in young plant tissues, including flowers and roots, and that *BpJMJ20/22* is mostly expressed in germinating seeds, as confirmed by qRT-PCR. Moreover, a detailed expression profile revealed that cold stress affects most *BpJMJ* genes, of which those with LTR elements will be down-regulated in response to the cold stress. Finally, the gene co-expression network (WGCNA) and GO enrichment analysis reveal the main way in response to the low-temperature condition. Both of these findings, taken together, will serve as a critical foundation for future studies on the role of particular *BpJMJ* genes and research on histone methylation and demethylation in birch.

## Methods

### Plant materials and seed germination

The birch seeds were acquired from the State Key Laboratory of Tree Genetics and Breeding (TGB) at Northeast Forestry University, China, in 2020. The seeds were sown into plastic pots of 25 cm diameter and 35 cm height in June and grown in the greenhouse for 3 months. Subsequently, the roots, stems, and leaves were collected, frozen, and stored in liquid nitrogen for subsequent experimental analysis.

In the seed germination experiment, the newly collected seeds were wholly first immersed in a 10% phosphate buffer saline (PBS) buffer, shaken for 2 days, and then allowed to stand for a week in the dark 4 °C. After that, seeds were washed with distilled water three times, 1 min each, and evenly spread on the sterilized soaked filter paper, store in a constant temperature 22 °C, 16 h light/8 h dark incubator. After ten days, the seeds that had just grown out of bacon and ungerminated seeds were both collected and stored in liquid nitrogen for subsequent experiments.

### Identification of **JmjC** domain-containing proteins in birch

To identify all potential *JHDM* homologous gene family in the *Betula pendula* genome (https://genomevolution.org/ CoGe/GenomeInfo.pl?gid=35079 and https://genomevolution.org/CoGe/ GenomeInfo.pl?gid=35080), the amino acid sequences of the *JHDM* genes determined in Arabidopsis (21), rice (20) and maize (19) (Table S13), were used to query the protein sequence database of *Betula pendula* genome with blastp program (*P*-value <1e-10). The JmjC domains (Pfam: PF02373, SMART: SM00558) [68, 69] were used to filter the candidates by HMM search engine (http://www.hmmer.org) [70]. Subsequently, the presence of JmjC domains in the candidate’s protein was verified by CDD https://www.ncbi.nlm.nih.gov/ (https://www.ncbi.nlm.nih.gov/Structure/cdd/wrpsb.cgi) [71, 72] and SMART (http://smart.embl.de/) [73]. Consequently, 21 homologous *BpJMJ* genes were confirmed in birch genome after removing redundant transcripts, which are uniformly named as *BpJMJ1-BpJMJ21* based on their genomic localization.

The basic information of the *JHDM* genes in birch, including the gene ID, CDS, and amino acid number, exon and intron location, and the physical location, are all extracted and calculated from the genome annotation. The isoelectric point (PI) and molecular weight (kDa) of the genes are obtained through ExPASy (http://www.expasy.org/tools/) website using default parameters [74]. The motif and functional domain of the *BpJMJ* genes are predicted from the MEME (https://meme-suite.org/meme/) and PFAM websites, respectively [75, 76]. In addition, the homology alignment of the *BpJMJ* gene with other species is performed on the Jalview (http://www.jalview.org/getdown/release/) with muscle algorithm.

### Phylogenetic analysis of **JHDM** proteins in birch

The amino acid sequences of the JHDMs in Arabidopsis, rice, and maize are obtained from Qian et al. 2018 and Cao et al. 2008. The newly predicted JHDM proteins in birch were aligned with JHDM members in Arabidopsis, rice, and maize-based on Muscle algorithm in MEGA6.0 (https://www.megasoftware.net). Then, the phylogenetic relationship among these four species was calculated with the neighbor-joining method and 1000 bootstrap corrections and visualized in the iTOL (https://itol.embl.de) [77]. The protein sequences involved in the phylogenetic tree and the Newick file obtained after MEAG software analysis are provided in the Table S14, and Table S15.

### Chromosomal localization, synteny analysis, gene duplication events, and Ka/Ks analysis of *JHDM* genes in birch

The physical location of 21 *JHDM* genes in birch was obtained from the genome annotation, and TBtools mapped their distribution in each chromosome. The gene duplication of *JHDM* genes was characterized using BLASTp and Multiple Collinearity Scan toolkit (MCScanX) (http://chibba.pgml.uga.edu/mcscan2/#tm) [78], and the synteny analysis of *JHDM* genes among the *Arabidopsis thaliana, Glycine max, Populus, Oryza sativa*, and *Zea mays* was performed in TBtools with default parameters. The protein data for Arabidopsis is downloaded from TAIR website (https://www.arabidopsis.org), and the protein data for the other species is extracted from Esemble Plants (http://plants.ensembl.org/index.html).

The Ka value, Ks value, and Ka/Ks ratios for the paralogs *JmjC* gene-pairs were calculated by TBtools. The paralogous genes were identified by searching the term “syntenic region” in the birch genome. The rate of divergence was calculated by using the following formula: T = Ks/2r, where Ks represents the synonymous substitutions per site and r is the rate of divergence. For dicotyledonous plants, the hypothesis is 1.5 synonymous substitutions per site of 10^8^ years [79].

### Identification of cis-acting elements of $JHDM$**genes** promoter

The 2 kb upstream genomic DNA sequence (promoter region) of the 21 *JHDM* family gene was retrieved from the birch genome by TBtools and submitted to the PlantCare website (http://bioinformatics.psb.ugent.be/webtools/plantcare/html/) for prediction of the cis-acting elements. The 13 most frequent cis-acting elements were visualized in TBtools [80].

### The tissue-specific transcriptome profile of the *JHDM* gene family

The RNA-seq sample datasets for each tissue, including leaf, root, flower, and xylem, were obtained from two-year-old birch at the Experimental Station of Northeast Forestry University, Harbin, P.R. China. Sequencing data were provided in Gene Expression Omnibus under accession number PRJNA535361. The bioinformatics analysis process is as follows: a) the original transcriptome data was aligned to the birch genome with hisat2 (https://daehwankimlab.github.io/hisat2/). b) the aligned results were performed to calculate TPM (Transcripts Per Million) by stringtie (http://ccb.jhu.edu/software/stringtie/). c) Finally, the *BpJMJ* gene expression in different tissues was scaled by setting the scale = “row” parameter in pheatmap function of R (https://cran.r-project.org), and then the hierarchical clustering of expression profiles of 21 *BpJMJ* genes was visualized by R-heatmap package.

### The expression analysis of the ***JHDM*** gene family under abiotic stress

The high-throughput transcriptome sequencing of birch treated with low temperature (6 degrees Celsius) during five periods (0.5 h, 1 h, 1.5 h, 2 h, 2.5 h, and 3 h) was downloaded from Yan et al. (2020), [81], in which the control was planted in an environment of 25 °C without low-temperature treatment. Sequencing data were provided in Gene Expression Omnibus under accession number PRJNA532995, and the bio-informatics procedure was followed above.

With the aim of identifying the *JHDM* genes in response to salt in birch, transcriptome libraries from the leaves of control and plants with 200 mM NaCl treatment were provided by Shao et al. (2018) [82]. Sequencing data were provided in Gene Expression Omnibus under accession number PRJNA471213, and the bio-informatics procedure was followed above.

### Genomic RNA extraction and quantitative RT-PCR analysis

The roots, stems, leaves collected from the three-month-old birch seedlings cultivated in the soil, ungerminated and germinating seeds, were ground as a fine powder by liquid nitrogen. Total RNA was isolated with Eastep® Super (Promega Beijing) according to the manufacturer’s instructions. The specific primers of genes for qRT-PCR were designed according to the birch genome and Geneious (http://www.geneious.com), which are list in Table S16. About 1 μg RNA was mixed with the 10 ul reverse-transcribed buffer in 2 ml PCR-tube, which comprising 1 ul gDNA Eraser Buffer, 2 ul 5×g DNA Eraser Buffer and the rest is RNase free ddH_2_O. The standard protocol was set as 2 min at 42 °C, followed by 10 min at 0 °C. The reaction product was added to a 10 ul reaction containing 1 ul Primer Script RT Enzyme, 1 ul RT Primer Mix, 4 ul 5 × Primer Script Buffer, and RNase Free ddH2O. The reverse transcribes parameters were as follows: 15 min at 37 °C followed by 5 s at 85 °C and 10 min at 0 °C. The ten-fold diluted digestion DNA products were used as a template for quantitative RT-PCR of betulin synthetic genes. The *tubulin* gene was identified from the birch reference genome as the internal control. PCR analysis was conducted using the Applied ABI7500 Real-Time PCR System with SYBR Premix Ex Taq™ II. Each PCR was conducted in a 15 ul reaction mixture containing 6.3 ul of 20 X diluted cDNA, 7.5 ul of SYBR Green Supermix, 0.6 ul of forward primers, and 0.6 ul of reward primers. Each gene of transcript level was calculated by the 2-(−ΔΔCt) CT method. All the qRT-PCR experiments were performed in three independent replicates.

### Co-expression network construction and GO enrichment analysis of $BpJMJ$ gene family

The WGCNA (Weighted Correlation Network Analysis) https://horvath.genetics.ucla.edu/html/ (https://horvath.genetics.ucla.edu/coexpressionnetwork/) [83] were obtained to identify co-expressed genes with the low-temperature responsive *BpJMJ* genes and was visualized in Cytoscape (https://cytoscape.org/) [84]. The data used as input for the co-expression network construction are the TPM derived from the time series experiment of low-temperature stress. Only genes with a TPM greater than 10 in all samples are retained. The optimalβ (soft thresholding power) value was determined to be 9 after 20 iterations. The Pearson algorithm is then used to calculate the correlation coefficient, and the result is stored as a signed co-expression matrix. The parameter “minModuleSize” value was set to 30, and the parameter “mergeCutHeight” value was set to 0.15. To ensure the reliability and readability of the results, the edges were filtered with weights below 0.1 and used Cytoscape to display genes directly/indirectly related to *BpJMJ* genes. The GO annotation was performed by goatools (https://github.com/tanghaibao/goatools) [85] with the fisher-exact test algorithm and visualized using R.

## Supplementary Information

**Additional file 1: Table S1**. Basic information of *JHDM* genes identified in birch.

**Additional file 2: Table S2**. Homolog similarity of *JHDM* genes between birch and Arabidopsis.

**Additional file 3: Table S3**. The MEME motif anotation of JHDM members in birch.

**Additional file 4: Table S4**. The distribution of functional domains in different JmjC subfamilies of birch.

**Additional file 5: Table S5**. The classification of duplicate events in *JHDM* genes subfamily.

**Additional file 6: Table S6**. Ka/Ks calculation of the paralog pairs of *BpJMJ* in birch.

**Additional file 7: Table S7**. Expression levels (TPM) of *JHDM* genes in different birch tissues.

**Additional file 8: Table S8**. The spearman correlation between the number of *BpJMJ* genes introns and their expression in different tissues.

**Additional file 9: Table S9**. The spearman correlation between the number of *BpJMJ* genes cis-acting elements and their expression in different tissues.

**Additional file 10: Table S10**. The quantitative distribution of cis-acting elements in the promoter region of *BpJMJ* gene family.

**Additional file 11: Table S11**. Expression levels (TPM) of *JHDM* genes with cold stress.

**Additional file 12: Table S12**. Expression levels (TPM) of *JHDM* genes with salt stress.

**Additional file 13: Table S13**. *JHDM* gene distribution among Birch, Maize, Arabidopsis and Rice.

**Additional file 14: Table S14**. The BpJMJ protein sequences of Arabidopsos, birch, rice and may involved in the phylogenetic tree.

**Additional file 15: Table S15**. The Newick file obtained from MEGA software for constructing the phylogenetic tree.

**Additional file 16: Table S16**. The primers for qRT-PCR used in this study.

**Additional file 17: Figure S1**. The detailed sequences information of 20 motifs in BpJMJ proteins annotated from MEME website.

**Additional file 18: Figure S2**. Protein sequence alignments of KDM5/JARID1 and KDM4/JHDM3 subfamily among birch, Arabidopsis, rice and may.

**Additional file 19: Figure S3**. Protein sequence alignments of KDM3/JHDM2, JMJD6 and JmjC domain-only subfamily among birch, Arabidopsis, rice and may.

**Additional file 20: Figure S4**. Distribution of cis-acting elements (hormone, light and stress) in 2kb promoter region of 21 *BpJMJ* genes.

**Additional file 21: Figure S5**. The count of *BpJMJ* genes with different classification of cis-acting elements.

**Additional file 22: Figure S6**. The expression of *BpJMJ* genes analyzed by transcriptome in response to salt treatment (200mM NaCl).

**Additional file 23: Figure S7**. Different gene modules of birch under cold stress analyzed by WGCNA.

**Additional file 24: Figure S8**. The co-expression network (network1) for the low-temperature response *BpJMJ* genes.

**Additional file 25: Figure S9**. GO enrichment (MF) analysis of *BpJMJ* directly associated genes in co-expression network.

## Data Availability

All transcriptome data, including **PRJNA535361** (https://www.ncbi.nlm.nih.gov/bioproject/PRJNA535361), **PRJNA532995** (https://www.ncbi.nlm.nih.gov/bioproject/PRJNA532995) and **PRJNA471213** (https://www.ncbi.nlm.nih.gov/bioproject/PRJNA471213) used and/or analyzed during the present study are available in NCBI.

## Abbreviations

ABRE: Cis-acting element involved in the abscisic acid responsiveness
W box: Elicitation
TGA-element: Auxin-responsive element
ERE: Ethylene-responsive element
TCA-element: Cis-acting element involved in salicylic acid responsiveness
CGTCA-motif: Cis-acting regulatory element involved in the MeJA-responsiveness
G-box: Light responsiveness
GT1-motif: Light responsiveness
ARE: Anaerobic induction
TGACG-Motif: cis-acting regulatory element involved in the MeJA-responsiveness
TCT-motif: Light responsiveness
Box 4: Light responsiveness
LTR: Low-temperature responsiveness;
