# Supplementary material for: Genome-wide identification, classification, and expression analysis of the JmjC domain-containing histone demethylase gene family in birch
Source: BMC Genomics. 2021 Oct 28;22:772. doi: 10.1186/s12864-021-08063-6 (PMC8555302; doi:10.1186/s12864-021-08063-6)
Supplement: Supplementary file 17 — Additional file 17: Figure S1. The detailed sequences information of 20 motifs in BpJMJ proteins annotated from MEME website. [file 12864_2021_8063_MOESM17_ESM.pdf]

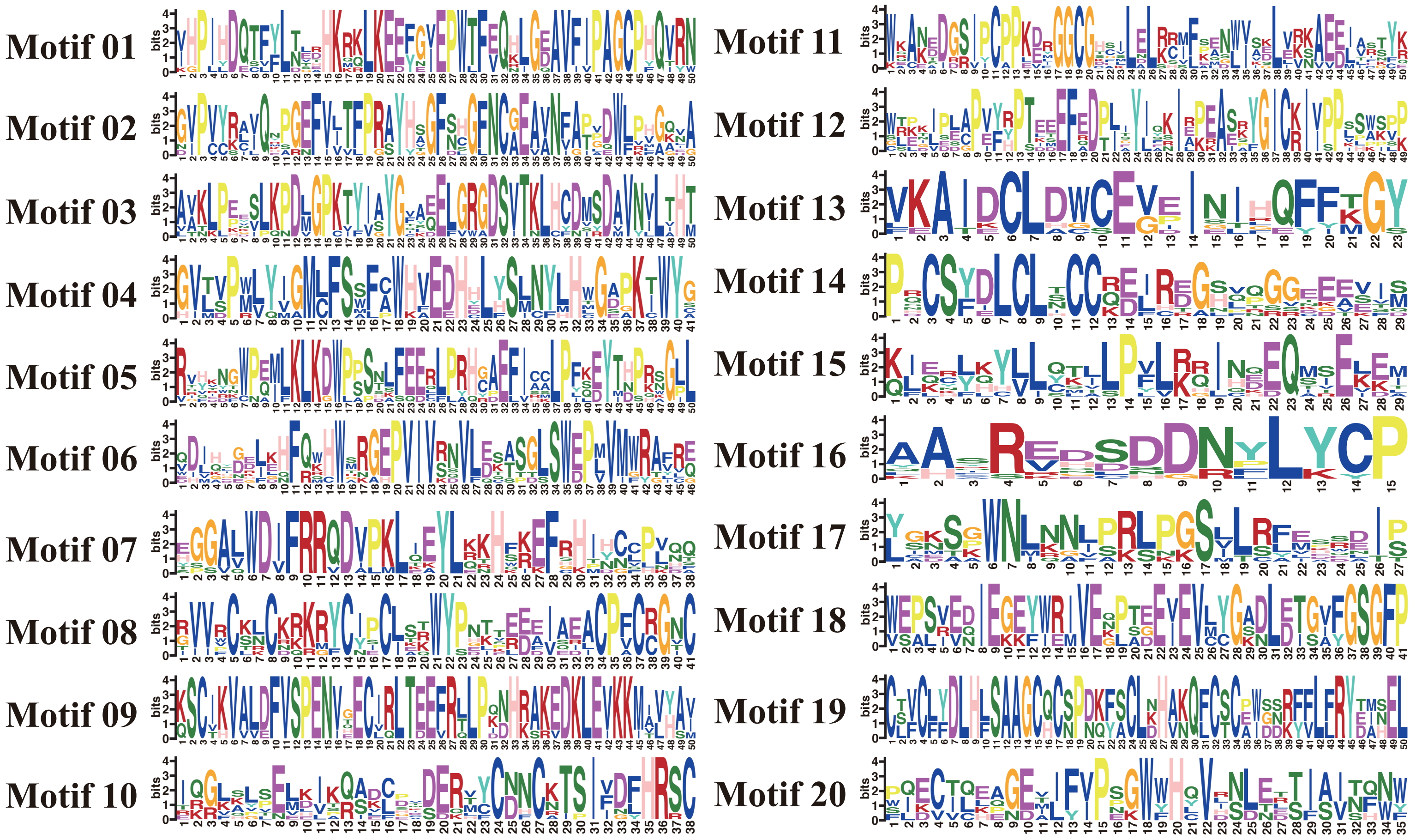

Figure S1. The detailed sequences information of 20 motifs in BpJMJ proteins annotated from MEME website.
