# Supplementary figures and images for: Genome-wide identification, classification, and expression analysis of the JmjC domain-containing histone demethylase gene family in birch
Source: BMC Genomics. 2021 Oct 28;22:772. doi: 10.1186/s12864-021-08063-6 (PMC8555302; doi:10.1186/s12864-021-08063-6)

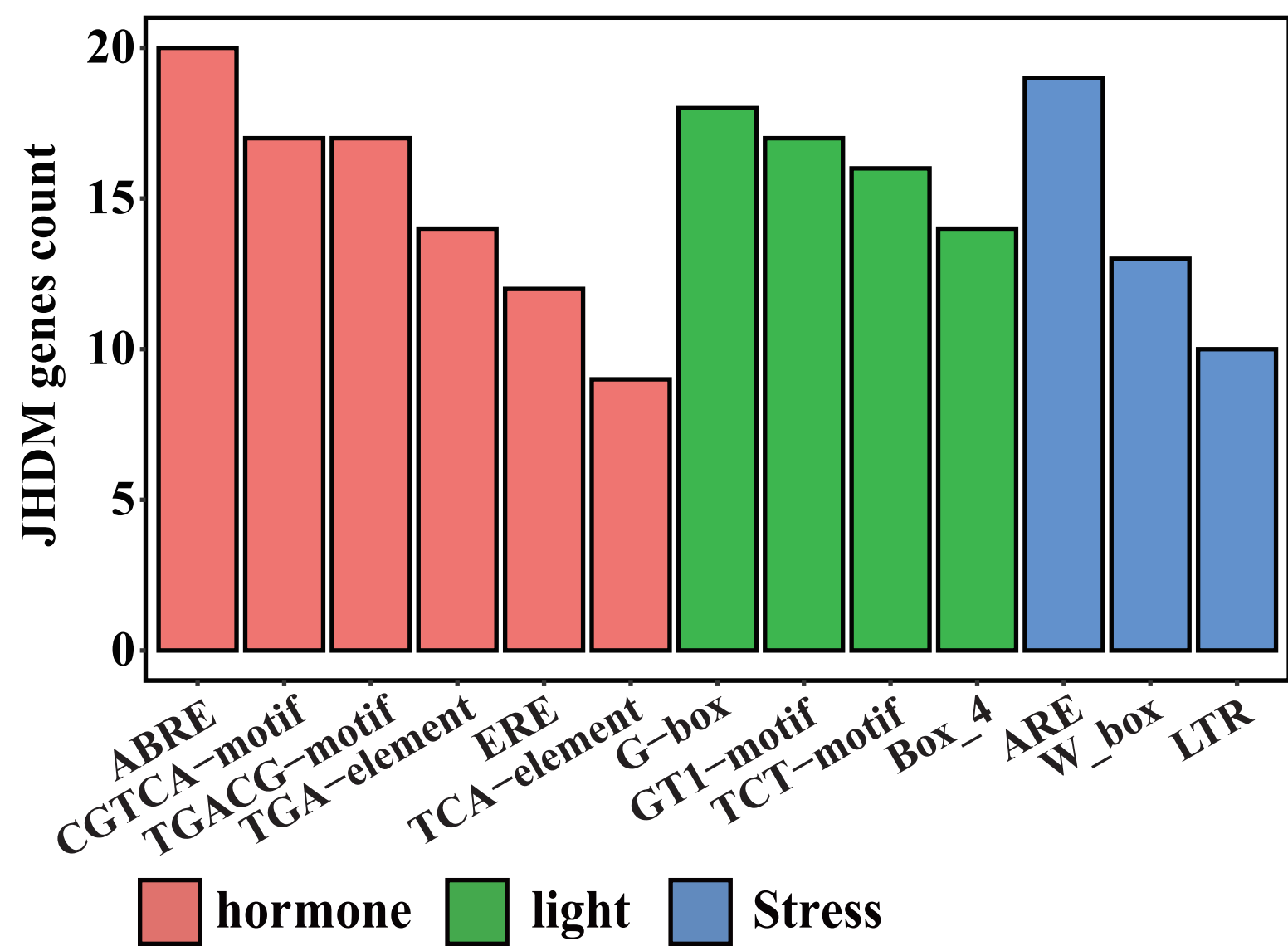

**Figure S5.** The count of *BpJMJ* genes with different classification of cis-acting elements.

Supplement: Supplementary file 21 — Additional file 21: Figure S5. The count of BpJMJ genes with different classification of cis-acting elements. [file 12864_2021_8063_MOESM21_ESM.pdf]
