# Supplementary material for: Genome-wide identification, classification, and expression analysis of the JmjC domain-containing histone demethylase gene family in birch
Source: BMC Genomics. 2021 Oct 28;22:772. doi: 10.1186/s12864-021-08063-6 (PMC8555302; doi:10.1186/s12864-021-08063-6)
Supplement: Supplementary file 22 — Additional file 22: Figure S6. The expression of BpJMJ genes analyzed by transcriptome in response to salt treatment (200mM NaCl). [file 12864_2021_8063_MOESM22_ESM.pdf]

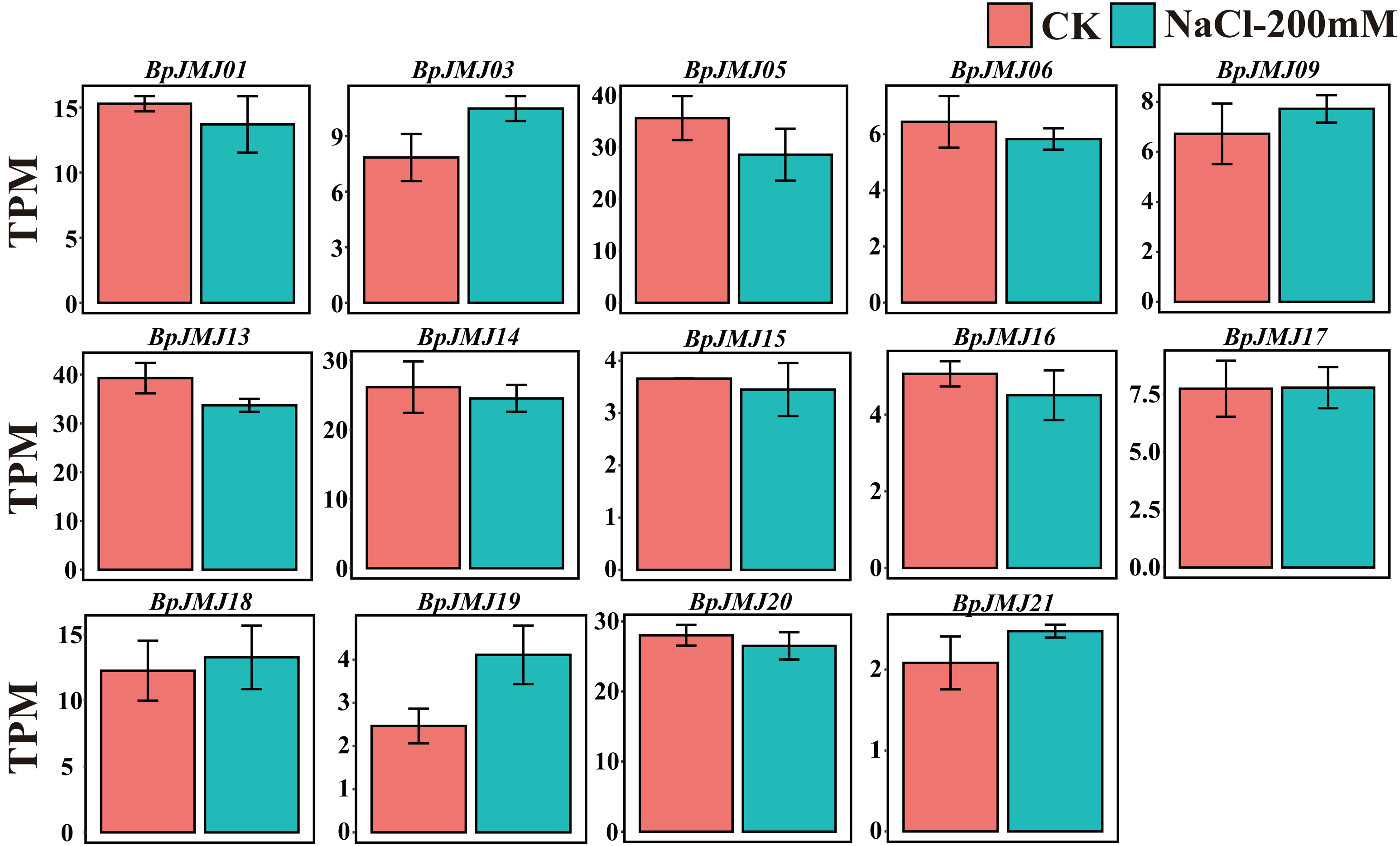

Figure S6. The expression of *BpJMJ* genes analyzed by transcriptome in response to salt treatment (200mM NaCl).
