# Supplementary material for: Genome-wide identification, classification, and expression analysis of the JmjC domain-containing histone demethylase gene family in birch
Source: BMC Genomics. 2021 Oct 28;22:772. doi: 10.1186/s12864-021-08063-6 (PMC8555302; doi:10.1186/s12864-021-08063-6)
Supplement: Supplementary file 25 — Additional file 25: Figure S9. GO enrichment (MF) analysis of BpJMJ directly associated genes in co-expression network. [file 12864_2021_8063_MOESM25_ESM.pdf]

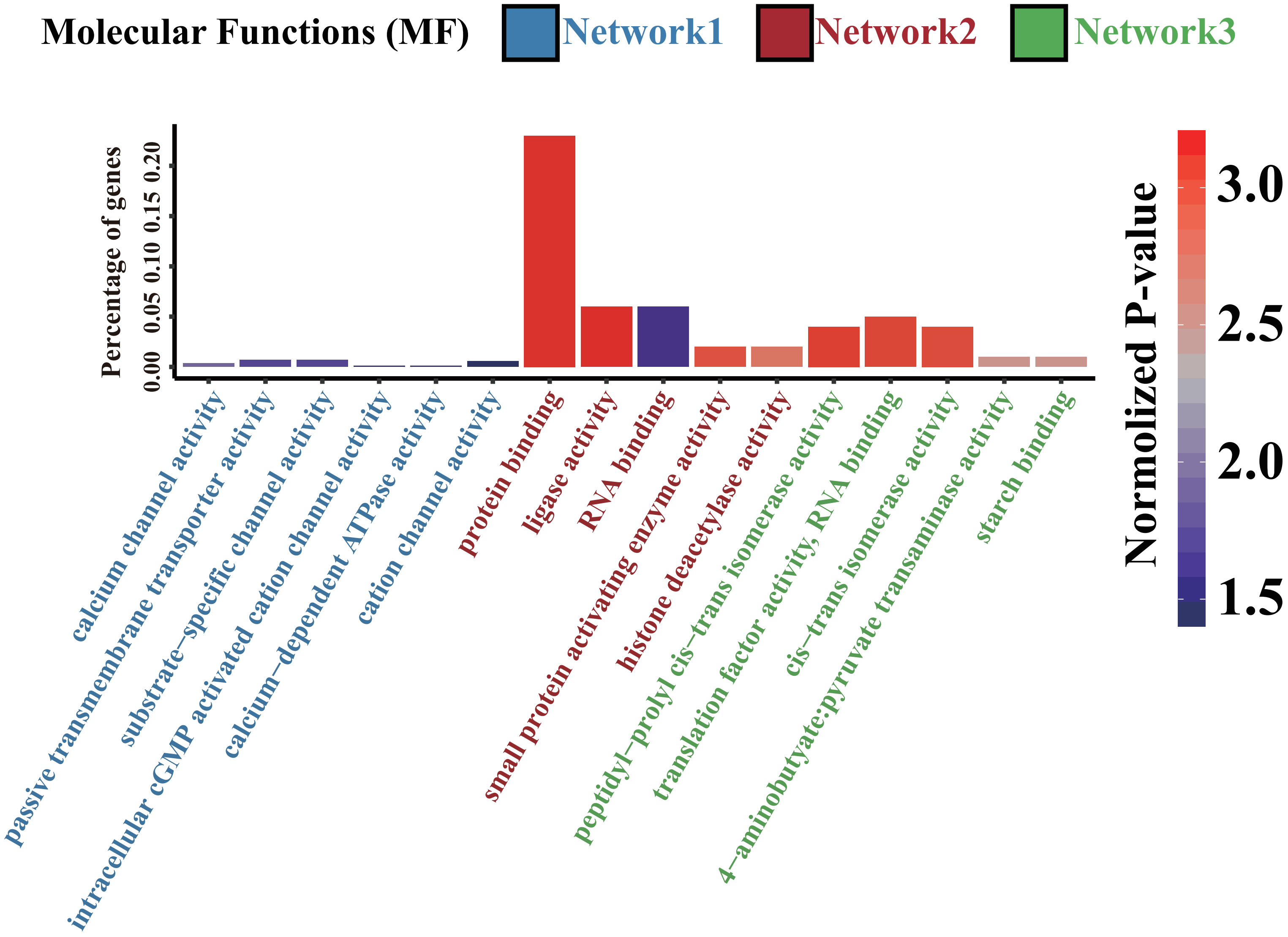

Figure S9. GO enrichment (MF) analysis of *BpJMJ* directly associated genes in co-expression network.
